# Identification of a novel RHO heterozygous nonsense mutation in a Chinese family with autosomal dominant retinitis pigmentosa

**DOI:** 10.1186/s12886-021-02125-9

**Published:** 2021-10-11

**Authors:** Wei Liu, Ruru Guo, Huijie Hao, Jian Ji

**Affiliations:** Tianjin Key Laboratory of Retinal Functions and Diseases, Tianjin Branch of National Clinical Research Center for Ocular Disease, Eye Institute and School of Optometry, Tianjin Medical University Eye Hospital, Tianjin, 300384 China

**Keywords:** Retinitis pigmentosa, RHO gene, Rhodopsin

## Abstract

**Background:**

To explore the molecular genetic cause of a four-generation autosomal dominant retinitis pigmentosa family in China.

**Methods:**

Targeted region sequencing was performed to detect the potential mutation, and Sanger sequencing was used to validate the mutation. Multiple sequence alignment from different species was performed by CLUSTALW. The structures of wild-type and the mutant RHO were modeled by Swiss-Model Server and shown using a PyMOL Molecular Graphic system.

**Results:**

A novel heterozygous nonsense mutation (c.1015 A > T, p.Lys339Ter, p.K339X) within RHO, which cosegregated with retinitis pigmentosa phenotype was detected in this family. Bioinformatics analysis showed the mutation was located in a highly conserved region, and the mutation was predicted to be pathogenic.

**Conclusions:**

We identified a novel heterozygous nonsense mutation of RHO gene in a Chinese family with retinitis pigmentosa by target region sequencing and our bioinformatics analysis indicated that the mutation is pathogenic. Our results can broaden the spectrum of RHO gene mutation and enrich the phenotype-genotype correlation of retinitis pigmentosa.

## Background

Retinitis pigmentosa (RP) is a heterogeneous group of inherited progressive retinal degenerative conditions and a leading cause of irreversible blindness. Typical fundus changes of RP include attenuated arterioles, waxy pallor of the optic disc, and characteristic perivascular and midperipheral bone-spicule pigmentation, which is resulted from melanin-containing vesicles accumulation in the retinal vascular layer and around the Muller cells [1].

RP affects 1 in 3500 individuals worldwide and there are several inherited patterns of RP, including autosomal dominant (15-20% cases), autosomal recessive (20-25% cases), X-linked (10-15% cases) or even mitochondrial inheritance [2]. To date, more than 89 genes have been reported to cause non-syndromic RP [3], with RP-GTPase regulator gene (RPGR; 10-20% of cases) and rhodopsin gene (RHO; 8-10% of cases) being the most commonly involved genes [4–8].

In this study, by target region sequencing, we identified a novel RHO nonsense mutation (c.1015 A > T, p.Lys339Ter, p.K339X) in a Chinese RP family.

## Methods

### Patients

This study was approved by the medical ethics committee of Tianjin Medical University Eye Hospital. A four-generation Chinese family suffered from RP was recruited and written informed consent was obtained. A 5 ml venous blood sample of each enrolled participant was collected from antecubital vein puncture, drawn into an ethylenediamine tetraacetic acid (EDTA) sample tube and stored in − 20 °C refrigerator for further analysis.

### Target region capture and sequencing

The target region capture and next-generation sequencing (NGS) procedure was described previously [9]. Briefly, genomic DNA was firstly extracted according to the manufacturer’s standard procedure (MagPure Buffy Coat DNA Midi KF Kit, Magen, China). After that, the genomic DNA of the proband was sequenced with PE100 + 100 on MGISEQ-2000. Finally, the targeted sequences were captured using the NimbleGen SeqCap EZ Choice XL Library 24 Reaction 150217_HG19_CllnE_EZ_HX1 chip (Roche, Madison, USA) containing 156 genes related to retinal diseases (Table 1).

**Table 1:** The 156 retinal diseases-related genes enrolled in our targeted region sequencing

*ABCA4, ABHD12, ADAM9, ADGRV1, AIPL1, ALMS1, ARL2BP, ARL6, BBS1, BBS2, BEST1, BMP4, C2orf71, C8orf37, CA4, CABP4, CACNA1F, CACNA2D4, CAPN5, CDH23, CEP290, CERKL, CHM, CIB2, CLRN1, CNGA1, CNGA3, CNGB1, CNGB3, CNNM4, COL2A1, COL4A1, COL9A1, CRB1, CRX, CYP4V2, DHDDS, EFEMP1, ELOVL4, EMC1, ERBB3, EYS, FAM161A, FLVCR1, FSCN2, FZD4, GDF6, GNAT1, GNAT2, GPR179, GRK1, GRM6, GRN, GUCA1A, GUCA1B, GUCY2D, HARS, HGSNAT, HK1, IDH3B, IFT140, IFT172, IMPDH1, IMPG2, INPCDHR1, IQCB1, ITM2B, KCNJ13, KCNV2, KIF11, KLHL7, LCA5, LRAT, LRIT3, LRP5, MAK, MERTK, MFSD8, MVK, MYO7A, NDP, NEK2, NMNAT1, NR2E3, NRL, NYX, OFD1, OTX2, P3H2, P5E, PCDH15, PCYT1A, PDE6A, PDE6B, PDE6C, PDE6G, PDE6H, PEX1, PEX2, PEX5, PEX6, PEX10, PEX12, PEX13, PEX26, PITPNM3, PLA2G5, PNPLA6, POC1B, POMGNT1, PRCD, PROM1, PRPF3, PRPF6, PRPF8, PRPF31, PRPH2, RAB28, RAX2, RBP3, RBP4, RD3, RDH5, RDH12, RGR, RGS9, RGS9BP, RHO, RIMS1, RLBP1, RP1, RP2, RP9, RPE65, RPGR, RPGRIP1, SAG, SEMA4A, SIX6, SLC24A1, SNRNP200, SPATA7, TEAD1, TOPORS, TRNT1, TRPM1, TSPAN12, TTC8, TTLL5, TULP1, UNC119, USH1C, USH1G, USH2A, VCAN, ZNF513.*	

### Data analysis

To detect the potential variants in the family, we performed bioinformatics processing and data analysis after receiving the primary sequencing data. We used previously published filtering criteria to generate “clean reads” for further analysis [10]. The “clean reads” (with a length of 90 bp) derived from targeted sequencing and filtering were then aligned to the human genome reference (hg19) using the BWA (Burrows Wheeler Aligner) Multi-Vision software package [11]. After alignment, the output files were used to perform sequencing coverage and depth analysis of the target region, single-nucleotide variants (SNVs) and INDEL calling. We used GATK software [12] to detect SNVs and indels. All SNVs and indels were filtered and estimated via multiple databases, including NCBI dbSNP, HapMap, 1000 human genome dataset and database of 100 Chinese healthy adults. The Human Gene Mutation Database (HGMD, http://www.hgmd.cf.ac.uk/ac/index.php) was used to screen mutations reported in published studies.

### Sanger verification

All mutations and potential pathogenic variants were validated using conventional Sanger sequencing methods. Segregation analysis was performed in all available family members.

### Protein model construction

Multiple sequence alignment from different species was performed by CLUSTALW (https://www.genome.jp/tools-bin/clustalw). In addition, the structures of homomeric wild-type and the mutant RHO were modeled by Swiss-Model Server (https://swissmodel.expasy.org) and shown using a PyMOL Molecular Graphic system, using the solved structure of rhodopsin coded by RHO gene as template (Protein Data Bank No. 5W0P).

## Results

### Clinical evaluation

**Fig. 1:**
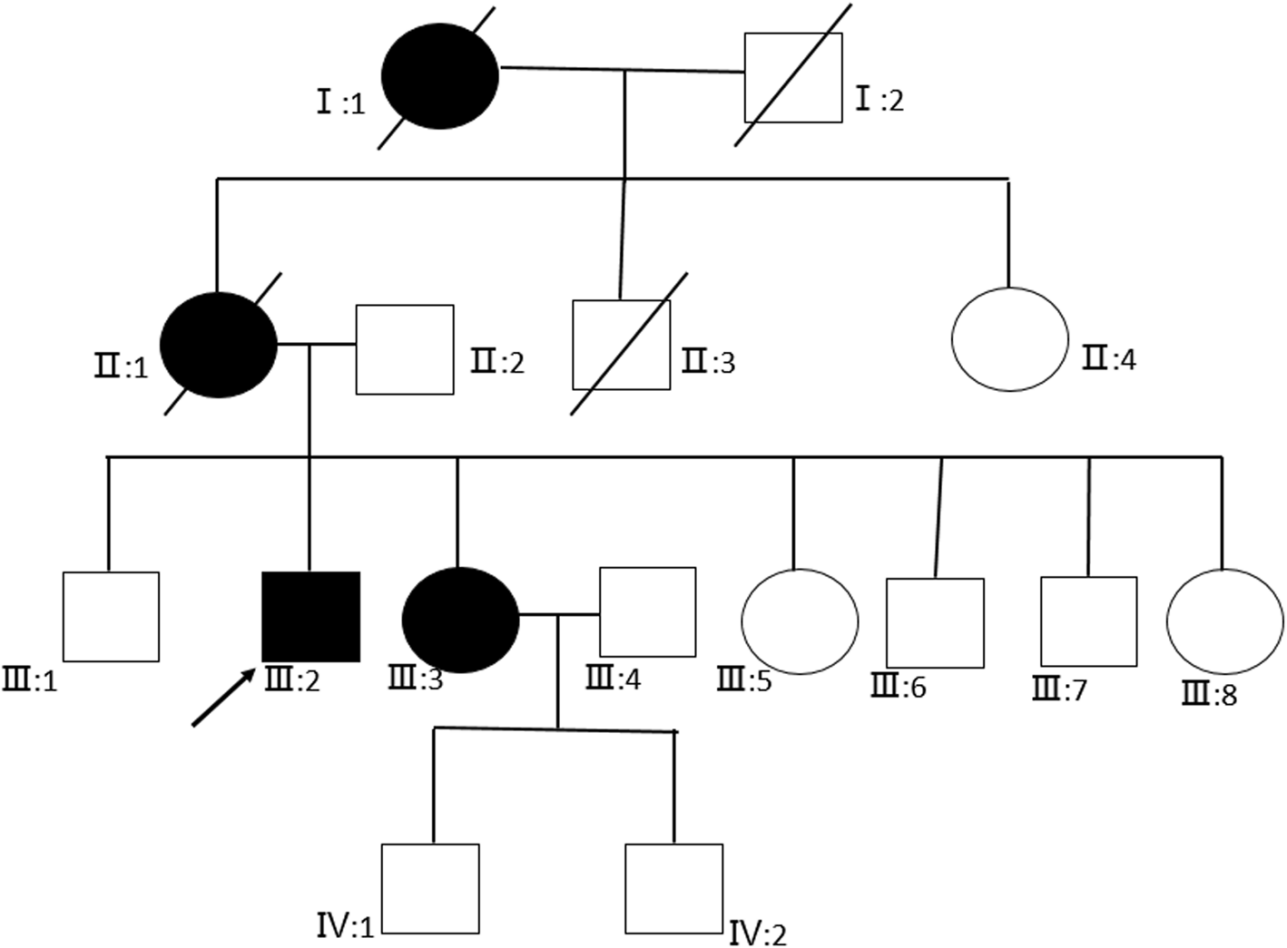
Pedigree map of the family. The arrow indicates the proband. Squares and circles symbolize males and females, respectively. Black and white denotes affected and unaffected individuals, respectively

**Fig. 2:**
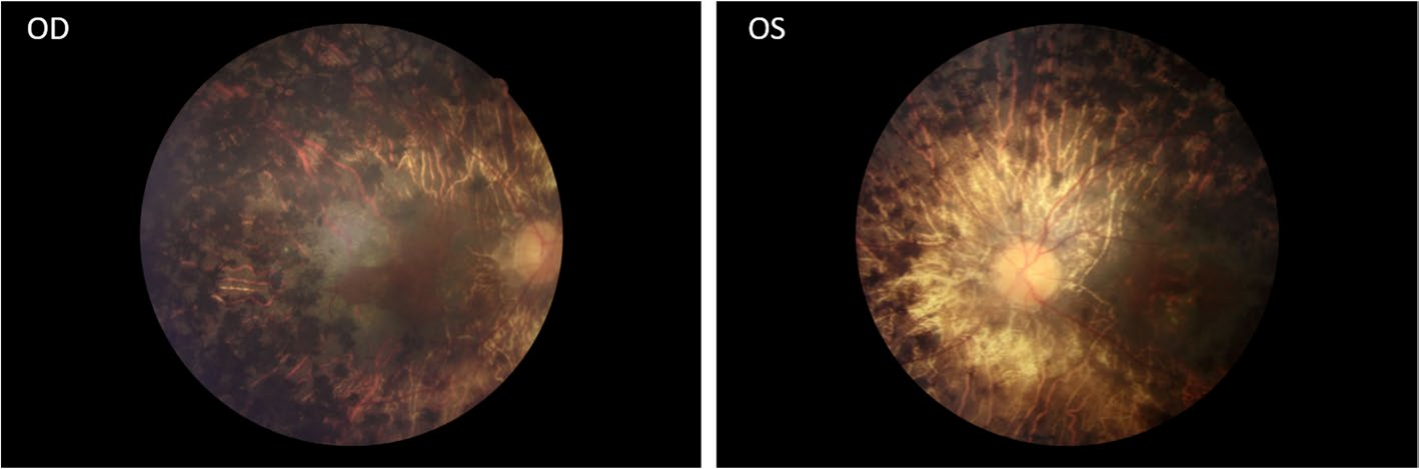
Fundus photographs of the proband. Note the attenuated arterioles, waxy pallor of the optic disc, atrophy of the choroid and diffuse bone-spicule pigmentation in both eyes

Two patients (III:2, III:3) and three normal individuals (III:4, III:5, IV:2) from this family were enrolled in this study (Fig. 1). The proband was a sixty-year-old man (III:2) and was diagnosed with RP when he was 17 years old. The patient suffered from night blindness when he was young and accepted cataract surgery 20 years ago. On presentation, the visual acuity of both eyes was light perception. The axial length was 25.99 mm and 25.56 mm, the central corneal thickness was 586 μm and 595 μm, the flat K was 42.56D and 43.27 D, the steep K was 43.80 D and 43.79 D, the intraocular pressure was 20.1 mmHg and 18.6 mmHg for the right eye and left eye, respectively. Under slit-lamp microscopy, the cornea was clear, the anterior chamber was deep and quiet, the pupil was sluggish and the intraocular lens was central and clear. Fundoscopic examination revealed attenuated arterioles, waxy pallor of the optic disc, atrophy of the choroid and diffuse bone-spicule pigmentation in both eyes (Fig. 2). The sister of the proband (III:3) also suffered from night blindness and was diagnosed with RP when she was 15 years old and she had similar fundus changes with the proband. There was no consanguineous marriage in this family.

### Mutation identification in RHO

**Fig. 3:**
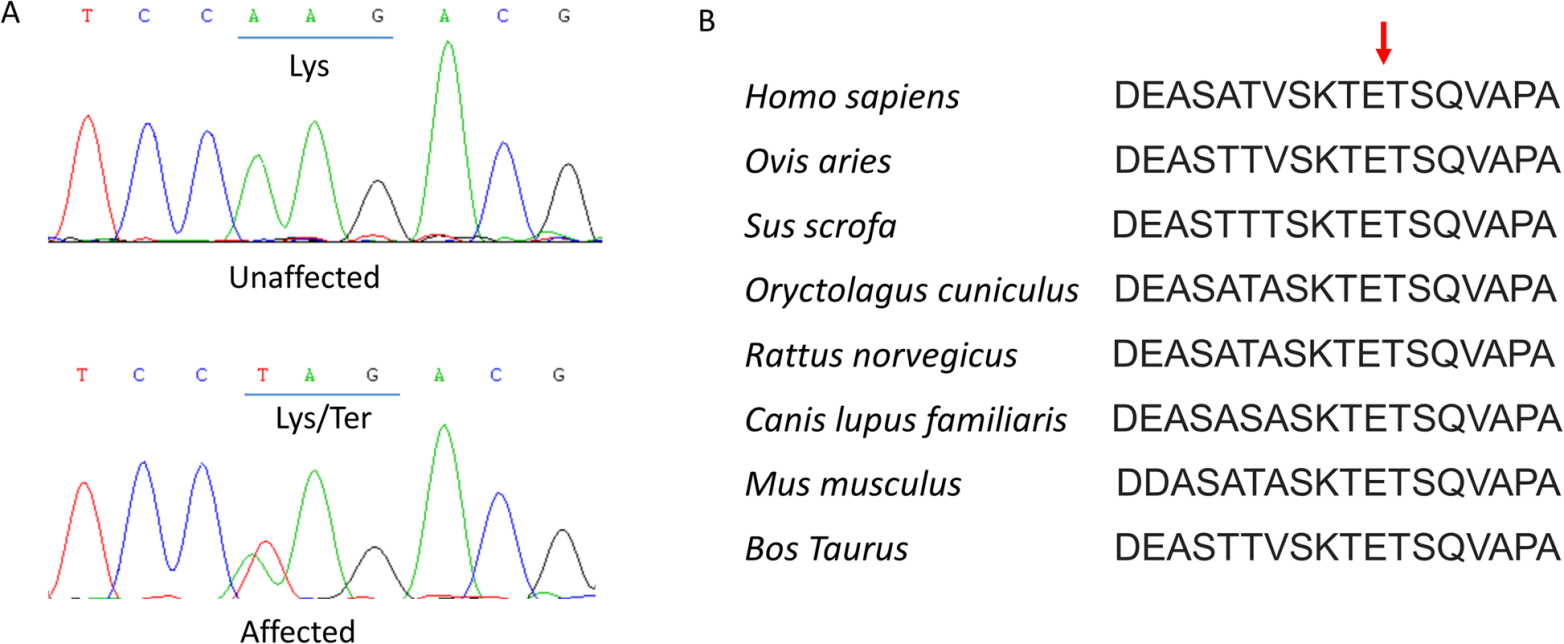
Sanger sequencing of RHO and bioinformatics analysis of the mutation. **A** Sanger sequencing of RHO detected a c.1015A > T transversion in affected patients which caused the replacement of a wild-type Lysine with stop codon at codon 339. **B** Multiple-sequence alignments of RHO in various species showed codon 339 was located within a highly conserved region

Targeted region sequencing containing 156 retinal diseases-related genes of the proband (III:2) revealed a transversion in exon 5 (c.1015A > T) of RHO, which was not reported previously. The variant causes the replacement of a wild-type Lysine with stop codon at codon 339 (p.Lys339Ter; Fig. 3A). The variant can be detected in all affected patients enrolled in this study (III:2, III:3) and was not found in the unaffected family member (III:4, III:5, IV:2) by further Sanger sequencing. The variant co-segregated with the disease in family members and was not found in NCBI dbSNP, HapMap, 1000 human genome dataset and database of 100 Chinese healthy adults, suggesting the variant may be the pathogenic mutation in this family.

### Protein model construction

**Fig. 4:**
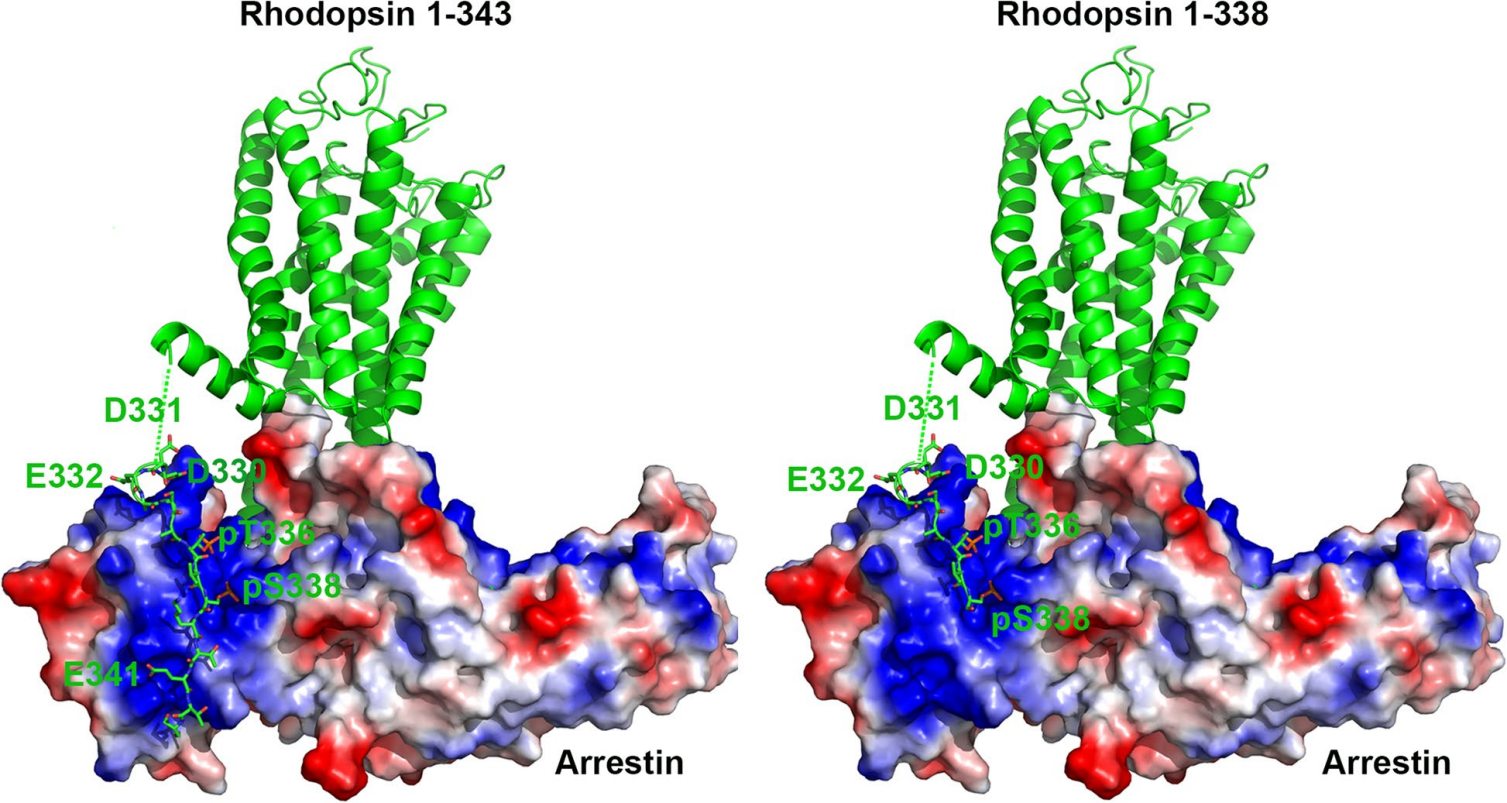
Structure model of rhodopsin-arrestin complex. Left: C-terminus of rhodopsin was shown in sticks, and the phosphorylation codes (D330, E332, T336, S338, E341) were highlighted. Arrestin was shown in vacuum electrostatics, blue area represents positive charge while red area represents negative charge. Normally, arrestin ‘reads’ phosphorylation codes of rhodopsin through code-sensing pockets. Right: K339X mutation of RHO produced a truncated protein and interfered the arrestin recruitment

Multiple-sequence alignments of RHO in various species showed codon 339 was located within a highly conserved region (Fig. 3B). Structure model of rhodopsin-arrestin complex showed normally, arrestin ‘reads’ phosphorylation codes of rhodopsin through code-sensing pockets in the C-terminus, while p.K339X mutation of RHO is predicted to produce a truncated protein and interfere with arrestin recruitment (Fig. 4).

## Discussions

In this study, we identified a RHO nonsense mutation (c.1015 A > T, p.Lys339Ter, p.K339X) in a Chinese RP family by target region sequencing and Sanger verification, which co-segregated with the disease in family members. This mutation appeared to be novel, which is neither reported in literature nor registered in HGMD. Our bioinformatics analysis showed codon 339 was located within a highly conserved region in various species and structure model showed the mutation could interfere arrestin recruitment, indicating this mutation was pathogenic and responsible for RP in this family.

RHO, located on chromosome 3q21-25, contains five exons. RHO was the first photoreceptor specific gene found to be mutated in autosomal dominant RP (adRP) and is the most common gene implicated in adRP. RHO gene accounts for 25-30% of adRP in Americans [13, 14], and the rate of RHO mutations in Asians is lower than in Americans [15–18]. The RHO gene encodes a 348-amino acids rod-specific protein rhodopsin, which is a typical G-protein-coupled receptor [19]. Rhodopsin localizes to the outer segment of rods and contains extracelluar, transmembrane, and cytoplasmic domains. RHO mutations often cause protein misfolding and retention in the endoplasmic reticulum, leading to cellular stress and eventual cell death [7].

Since the first p.P23H mutation in the RHO gene was discovered in 1990, more than 230 different mutations have been reported to be associated with RP in different ethnic populations, most of which are point mutations (HGMD). Based on their biochemical and cellular properties, these mutations can be grouped into class I or class II [20]. Class I mutations, located predominantly in the C-terminus of the protein, can fold normally, but are not correctly transported to the outer segment. Class II mutations, which usually occur in the intradiscal and transmembrane domains of the protein, are not folded correctly and are retained in the endoplasmic reticulum. Clinically, class I mutations lead to severe diffuse abnormal rod function early in life, while in class II mutations, the disease progresses slowly and the rods have nearly normal function even in adult patients.

Up to now, a total of 10 nonsense mutations have been reported in RHO (HGMD). It is suggested that the heterozygous truncating variants at the downstream of K296 could result in adRP [21]. The underlying mechanism for this may be that a truncated protein resulted from a heterozygous truncating variant after K296 could escape nonsense-mediated mRNA decay (NMD) and, thus, produce harmful truncated proteins. In contrast, the truncating variants before K296 are very close to benign variants, because the abnormal products from the truncating variants at the upstream of K296 will be cleared by the NMD process being triggered [21]. This is consistent with our results. The nonsense heterozygous mutation p.K339X detected in our study, located at the downstream of K296, led to adRP with severe phenotype.

It is recently reported that rhodopsin C-terminal tail served as an interface for arrestin recruitment, and the β strand (K339-T342) is one of the anchor points for rhodopsin-arrestin interaction [22]. Several mutations (Ala333Val; Thr340Met; Glu341Lys; Glu341Ter; Thr342Met; Ser343Asn; Ser343Cys; Gln344Pro; Gln344Arg; Gln344Ter; Val345Met; Val345Leu; Val345Gly; Ala346Pro; Pro347Thr; Pro347Gln; Pro347Arg; Pro347Leu; Pro347Ala; Pro347Ser; Pro347Cys; Ter349Gln; Ter349Glu, etc) have been reported in the C-terminal tail of rhodopsin (HGMD), indicating the C-terminus is a hotspot of mutation. The mutation detected in our study (p.K339X) is the first mutation found in codon 339, highlighting its role to maintain the normal function of rhodopsin. The mutation can interfere the arrestin recruitment and block the downstream signaling transmission, as shown in Fig. 4. As a class I mutation, p.K339X is expected to fold correctly but affect the post-Golgi trafficking of rhodopsin and impair its normal targeting to the photoreceptor outer segment [20], and lead to RP eventually.

## Conclusions

In conclusion, by target region sequencing, we identified a novel RHO nonsense mutation in a Chinese RP family. Our results can broaden the spectrum of RHO gene mutation, enrich the phenotype-genotype correlation of RP, and provide target for potential gene therapy in the future.

## Data Availability

All data generated or analysed during this study are included in this published article.

## Abbreviations

RP: Retinitis pigmentosa
RPGR: RP-GTPase regulator gene
RHO: Rhodopsin gene
EDTA: Ethylenediamine tetraacetic acid
NGS: Next-generation sequencing
SNVs: Single-nucleotide variants
HGMD: Human Gene Mutation Database
adRP: Autosomal dominant RP
NMD: Nonsense-mediated mRNA decay
